# A Habitat and a Parasite: Adult and Larval Parasitic Freshwater Mussels Impact Habitat Choice and Predator–Prey Interactions of a Host Fish and Its Prey

**DOI:** 10.1002/ece3.72601

**Published:** 2025-12-05

**Authors:** Sebastian L. Rock, Anna M. Elmlund, P. Anders Nilsson, Johan Watz, Olle Calles, Martin Österling

**Affiliations:** ^1^ Department of Environmental and Life Sciences, River Ecology and Management Karlstad University Karlstad Sweden; ^2^ Department of Biology—Aquatic Ecology Lund University Lund Sweden

**Keywords:** community ecology, conservation, ecosystem function, endangered species, freshwater, microhabitat, parasitism, Unionida

## Abstract

Parasitic freshwater mussels are endangered ecosystem engineers with an array of impacts on multiple trophic levels and life stages. While the impacts of adult mussels on separate trophic levels have been studied, few have directly tested how adult mussels can impact trophic interactions, or investigated the impacts of the parasitic mussel larvae (glochidia) on such interactions. We present a laboratory study which mimics two‐stream substrates for the endangered thick‐shelled river mussel ( *Unio crassus* ): one dominated by gravel and one by cobbles. First, the preference of a gammarid ( *Gammarus pulex* ) for mussel‐dominated habitats was tested in the presence/absence of chemical cues from the predator bullhead ( *Cottus gobio* ). Second, the preference of bullhead for mussel‐dominated habitats was tested under or without glochidia infestation. Third, the effect of infestation on bullhead predation on gammarids was assessed in the presence of adult mussels. Gammarids only significantly preferred mussel habitats in the absence of predator cues, whereas infested bullhead tended to prefer mussel habitats in cobble substrates. The presence of adult mussels only significantly reduced bullhead predation on gammarids in the gravel habitat, whereas infestation did not affect bullhead predation. Despite gammarids not preferring mussel habitat in the presence of predator cues, mussel beds represent valuable habitat to gammarids as mussel presence can facilitate a reduction of predation by bullhead. Infestation did not affect the rate of bullhead predation on gammarids but did attract bullhead to mussel habitat in cobble substrates. Our results suggest that mussel beds may be valuable habitat for both their host fish and the prey of their hosts, attracting both and increasing predator–prey interactions. This study highlights the cross‐trophic mechanisms by which multiple life stages of parasitic mussels can impact the interactions of their surrounding benthic community, underscoring their importance as ecosystem engineers.

## Graphical abstract

We tested the impact of adult and larval parasitic mussels on the predator–prey interactions between European native bullhead and gammarids. We demonstrate that the parasitic larvae of the mussels have a minimal effect on predation rates but adult mussels have a noteworthy impact on the habitat preference of both species.

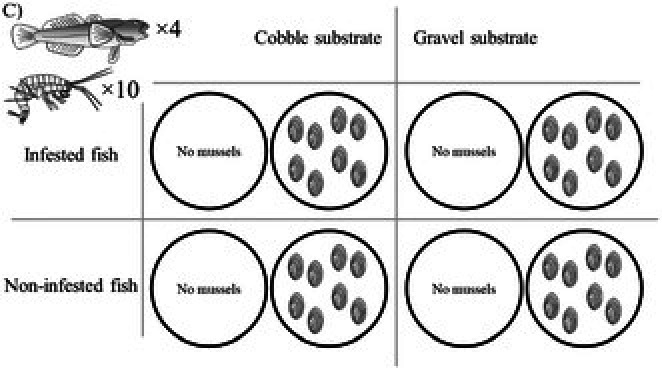

## 1 Introduction

Predator–prey interactions are fundamental ecological processes that shape community structure and drive evolutionary processes (Kerfoot and Sih 1987). Interest in these interactions has grown since their increased inclusion in studies on biodiversity and ecosystem function (Schmitz et al. 2017; van der Plas 2019). Predator–prey interactions are highly variable, and can be disrupted through changes to both biotic and abiotic factors (Weber et al. 2010; Heck and Crowder 1991; Laws 2017; Bastille‐Rousseau et al. 2018; DeBoom and Wahl 2013). Habitat complexity is among the most fundamental abiotic factors that have been shown to impact predator–prey interactions, typically as a function of microhabitat variability that increases overall biodiversity (Hansen 2000; Delclos and Rudolf 2011; Humphries et al. 2011; Hughes et al. 2012; Tokeshi and Arakaki 2012). As biodiversity increases, so too does the diversity and complexity of species interactions (Duffy et al. 2007).

Parasitism is a highly evolved species interaction which can have substantial effects on the behavior of the hosts, influencing both the predator and the prey in predator–prey interactions. Among the most well‐described effects of parasites on predator–prey interactions is parasite‐induced trophic transmission, where parasites alter the behavior of intermediate hosts to increase transmission to the next host, thereby regulating prey population density (Poulin 2010; Médoc and Beisel 2011; Hughes 2013). Conversely, parasites can reduce predator feeding rates, decreasing predation pressure on lower trophic levels and increasing prey density (Poulin 2007; Médoc and Beisel 2011; Filipsson et al. 2016). For directly transmitted parasites, transmission increases with host population density, often acting as a population regulator (Arneberg 2002; Mouritsen and Poulin 2005; Ryder et al. 2007; Wood et al. 2007; Friesen et al. 2018, 2020). Generally, as host behavior is altered, so is host habitat choice, which can have dynamic consequences on predator–prey interactions, particularly given their diverse impacts across trophic levels (Heuschele and Candolin 2010; Friesen et al. 2018; Brothers and Blakeslee 2021; Rock et al. 2025).

Freshwater mussels in the order Unionida are a globally endangered order of parasites, inflicting a disease known as glochidiosis on their host fish (Rock et al. 2022). Larval mussels (glochidia) affix onto a host, typically the gills of fish, for a growth and metamorphosis period, after which juvenile mussels excyst and fall to the sediment to then develop into adults (Strayer 2008). Generally, glochidiosis has a limited impact on their hosts, though it can reduce growth rates, increase metabolic rates and elevate mortality, particularly at high infestation rates (Douda et al. 2017; Filipsson et al. 2017; Chowdhury et al. 2019; Rock et al. 2022). Glochidiosis can also reorganize freshwater communities by decreasing host feeding rates (Crane et al. 2011; Österling et al. 2014), reducing swimming performance (Taeubert and Geist 2013; Filipsson et al. 2016) and altering habitat use (Horký et al. 2019; Rock et al. 2025) with some authors suggesting hosts may benefit from infestation (Ziuganov 2005; Marwaha et al. 2019; Chowdhury et al. 2021; Rock et al. 2022).

Unionids can additionally modify their surrounding community as ecosystem engineers, altering nutrient cycles, habitat structure, and food webs (Strayer et al. 2004; Vaughn et al. 2008; Vaughn and Hoellein 2018). As mussels filter particulates from the water column and egest material into the sediment, water clarity and sediment nutrient concentrations can become significantly higher, increasing both micro‐ and macrophyte production—boosting benthic and riparian biodiversity (Aldridge et al. 2007; Carroll et al. 2008; Allen et al. 2012; Atkinson et al. 2013; Chowdhury et al. 2016; DuBose et al. 2020; Benjamin et al. 2022). Mussel feces and pseudofeces (mixture of mucus and inorganic material excreted by mussels to remove non‐ingested filtered material) are both nutrient‐rich and are often fed on directly by benthic fauna (Sephton et al. 1980; Howard and Cuffey 2006; Vaughn et al. 2008; Garrido et al. 2012). Mussel beds can increase substrate surface roughness and habitat complexity, reducing water velocity, trapping suspended particles, and providing refuge for small organisms from high flow and predation, increasing biodiversity (Ziuganov et al. 1994; Stewart et al. 1998; Gutiérrez et al. 2003; Spooner et al. 2012; Bódis et al. 2014).

Unionid mussels have experienced critical population declines due to habitat loss, environmental degradation, and their parasitic life cycle depending on freshwater fish (Lopes‐Lima et al. 2017; Modesto et al. 2018)—many of their host fish are also endangered (Dudgeon et al. 2006). For example, European populations of the thick‐shelled river mussel ( *Unio crassus* ) have decreased dramatically in the second half of the 20th century alone (Lopes‐Lima et al. 2017). Motivated by our interest in the community‐wide effects of this endangered ecosystem engineer, we investigated the impact of *U. crassus* on predator–prey interactions between one of its common hosts, the European bullhead ( *Cottus gobio* ; Schneider et al. 2017) and a common gammarid amphipod prey ( *Gammarus pulex* ; Błońska et al. 2016). We first tested the preference for *U. crassus* habitats for both species in isolation: gammarids in the presence/absence of bullhead predator cues, and bullhead when infested with glochidia or not. We then tested the impact of glochidia on the bullhead‐gammarid predator–prey interaction in the presence and absence of adult *U. crassus* . In all studies, we additionally compared two substrate sizes: a gravel‐dominated substrate, with high refuge opportunity for gammarids and low surface roughness, where the mussel shells were the largest structures present, and a cobble‐dominated substrate, with low refuge opportunity for gammarids and high surface roughness, where the mussel shells were the smallest structures present. All three species investigated here are commonly found in both substrate types, and allowed us to compare the effects of *U. crassus* in multiple environments (Graça et al. 1994; Gosselin et al. 2010; von Proschwitz and Wengström 2021).

As gammarids typically seek refuge when exposed to predator cues (Dahl and Greenberg 1996; Ahlgren et al. 2011), and mussel presence increases habitat complexity (Gutiérrez et al. 2003; Bódis et al. 2014), we predicted that gammarids would generally prefer mussel habitats in the presence of predator cues. Our gravel‐dominated substrate offered more refuge opportunities for gammarids compared to our cobble‐dominated substrate given the greater number of interstitial spaces. We thus predicted that gammarids would prefer mussel habitats more in cobble‐dominated substrates, where mussel presence would have a greater impact.

As glochidiosis can reduce host fish swimming performance (Taeubert and Geist 2013; Filipsson et al. 2017; Slavík et al. 2017) and mussels typically increase surface roughness, we predicted that infested bullheads would generally prefer mussel habitats. Our cobble‐dominated substrates provided a higher surface roughness than our gravel‐dominated substrate. We thus predicted that infested bullheads would prefer mussel habitats more in the gravel‐dominated substrate, where mussel presence would have a greater impact.

Glochidiosis can also reduce host fish feeding rates (Österling et al. 2014; Filipsson et al. 2016), and we therefore additionally predicted that infested bullhead would generally predate less on gammarids in both substrate sizes. Our gravel‐dominated substrate offered a greater refuge opportunity for gammarids compared to our cobble‐dominated substrate. We thus predicted that predation rates would be lower in the gravel‐dominated substrate. As mussel presence typically increases habitat complexity (Gutiérrez et al. 2003; Bódis et al. 2014), we predicted that predation would be reduced in the mussel‐dominated cobble habitat, where the impact of mussel presence on habitat complexity would be stronger than in the gravel habitat.

## 2 Methods

### 2.1 General Laboratory Setup

This study was conducted at the “MusselLAB” in Hemmestorpsmölla, Sjöbo, Skåne, Sweden, between May and July 2023 with a standardized light regime of 12:12 (light: dark). Lab air temperature was maintained at 17°C ± 0.5°C with two portable air conditioning units (Model number: 013946, Anslut, Sweden), which maintained an average aquaria water temperature of 19.0°C ± 0.5°C. The water used in the lab was sourced from the nearby river Klingavälsån and was purified in‐house before use. Water purification started with a sequence of fine mesh filters (order and sizes: 200 μm, 100 μm, 50 μm, 10 μm, 5 μm, 5 μm, 1 μm, 1 μm), followed by an activated carbon filtration stage constructed from an EHEIM canister filter (Classic 500), equipped with three in‐line UV‐filters fitted with Philips TUV PL‐S 9 W lamps. Filtered water was then stored in a 500 L tank which underwent a two‐hour ozone treatment 18 h before use (Ozone generator model number: OAW03, Kinwodon, China).

**FIGURE 1:**
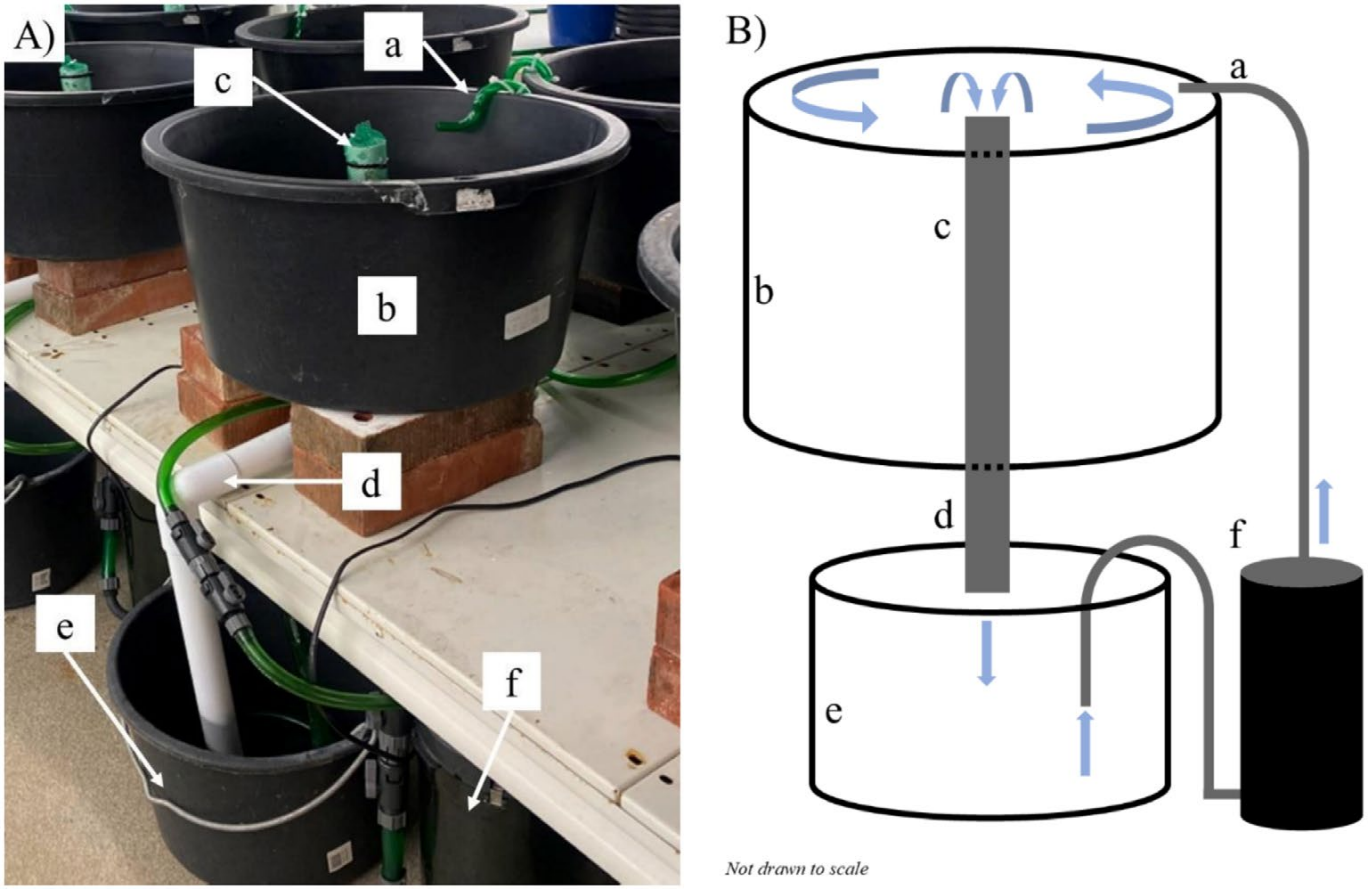
Image of one experimental unit (A) and graphical representation of one experimental unit (B; water flow indicated with blue arrows). Water flows from a directionally placed outlet (a) creating a circular flow in the experimental tank (b). Water then drains from a central standpipe (c) and drains (d) into a lower sump (e). An EHEIM canister filter (Classing 500) then pumps water back into the experimental tank (f).

**FIGURE 2:**
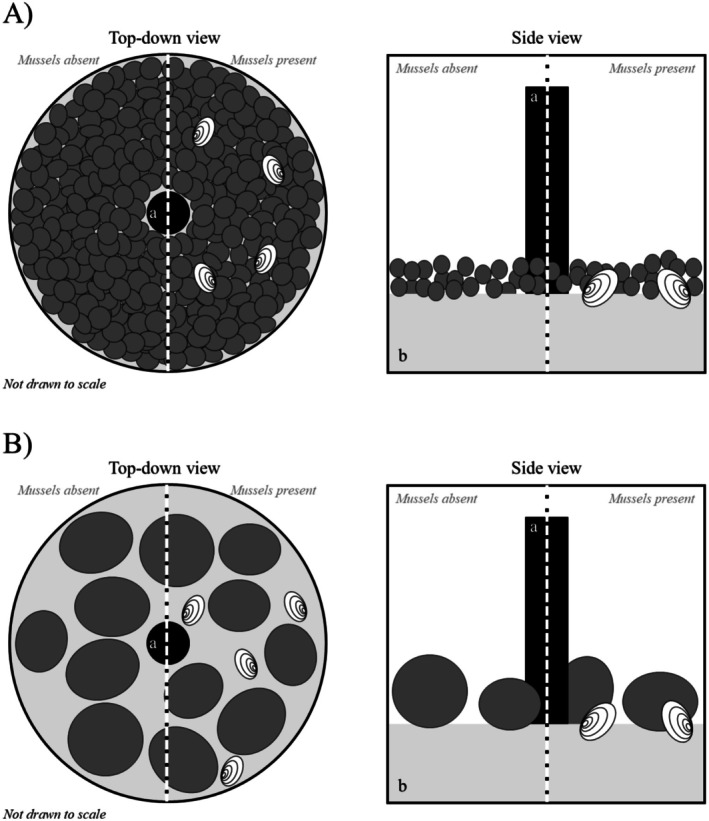
Graphical representation of the substrate treatments for the gravel‐dominated substrate (A) and the cobble‐dominated substrate (B). Central standpipe for the experimental unit indicated in black (a) and sandy bottom indicated in light gray (b). Dark gray ovals indicate gravel (size range 21–36 mm) in (A), and cobbles (size range 70–120 mm) in (B). Black and white dashed line separates a mussel‐dominated habitat (right) from a mussel‐absent habitat (left), mussels indicated with white and black ovals. Figures not drawn to scale.

All experiments were run in 12 circular, separately recirculating plastic tanks (Figure 1). Each tank consisted of a 40 L black plastic bucket (515 × 305 mm) with an overflow standpipe to set the water surface level and drain into a 20 L sump (380 × 255 mm). Water was returned to the experimental chamber with an EHEIM canister filter (Classic 500), with a directionally placed outflow to ensure a circular flow inside each tank. Each tank was filled with a 100 mm layer of pool filter sand (0.2–0.7 mm; Kayoba, Sweden). In addition, the tanks were filled with either 3 L of gravel (21–36 mm; ~200 pieces/tank) creating a permeable gravel bed 2–3 pieces deep (approximately 55 mm; Figure 2A), or 12 cobbles (70–120 mm) resting directly on the sand (Figure 2B).

The cobble‐dominated substrate had an estimated surface roughness of 95 mm (distance from the surface of the sand to the average height of the cobbles), whereas the gravel‐dominated substrate had an estimated surface roughness of 29 mm. The permeable gravel bed in the gravel‐dominated substrate had substantially more interstitial spaces for the gammarids to take shelter in than the bare sand bottom between the cobbles of the cobble‐dominated substrate; we thus refer to the gravel‐dominated substrate as being a more complex habitat than the cobble‐dominated substrate (Figure 2). By adding adult mussels to the cobble‐dominated substrate, we aimed to increase habitat complexity with a minimal impact on surface roughness, whereas by adding adult mussels to the gravel‐dominated substrate we aimed to increase surface roughness with a minimal impact on habitat complexity.

**FIGURE 3:**
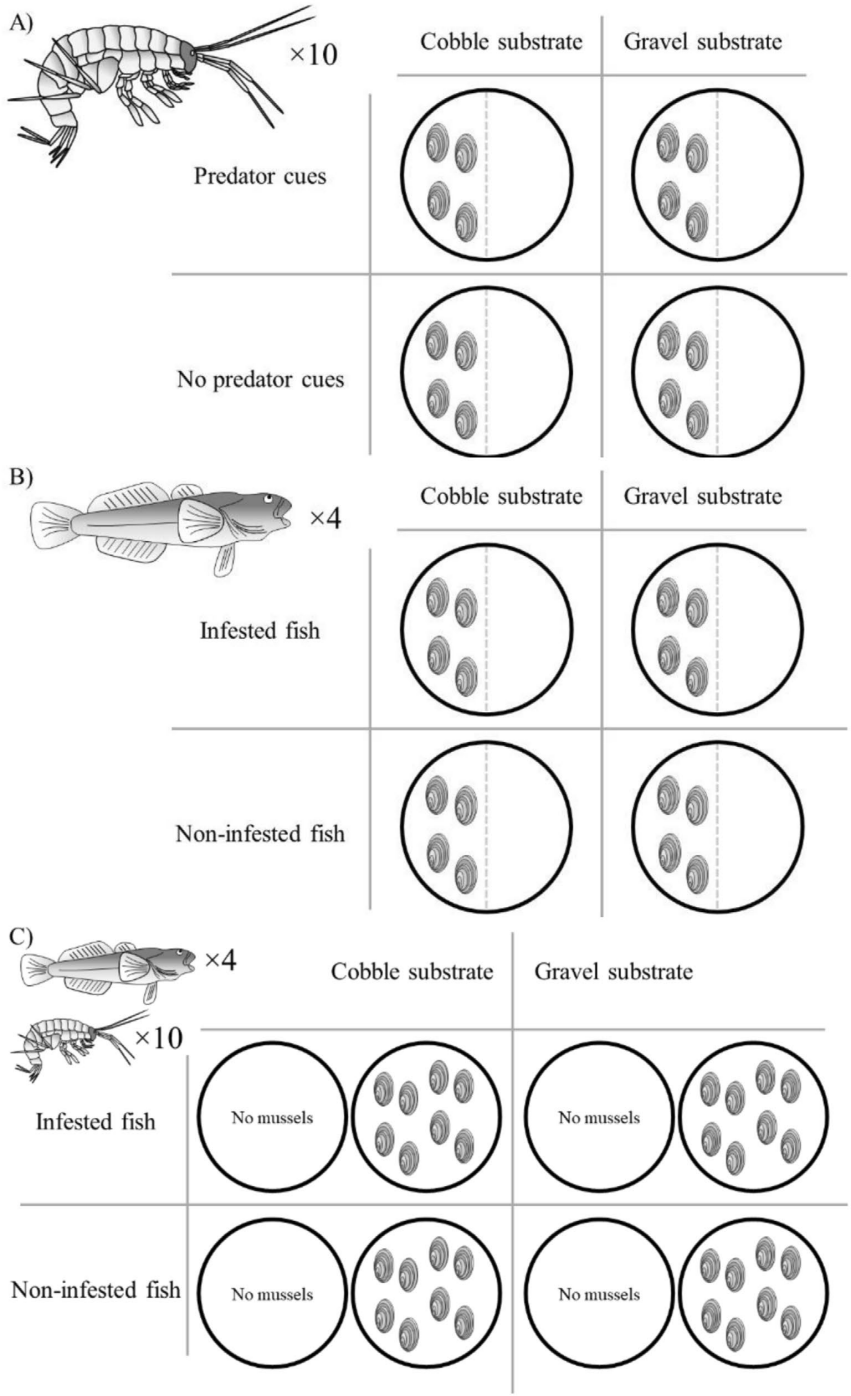
Graphical representation of the experimental design for the gammarid habitat preference study (A), bullhead habitat preference study (B), and predation study (C). Gray and black ovals indicate mussel presence, density, and placement for each experimental setup.

All mussel habitats used a mussel density of 19 mussels/m^2^, a density considered “high” in previous study (Schneider et al. 2019). For the gammarid and bullhead habitat preference experiments, four mussels were arranged in one half of each tank (Figure 3A,B). For the predation experiment, either eight or zero mussels were arranged in all tanks (Figure 3C). The two substrate sizes were additionally chosen to approximate the substrate sizes from which the animals were collected (see Section 2.2). Mussels and gammarids originated from sections of the river Vramsån primarily dominated by large stones on a sandy bed, whereas the bullhead originated from a section of the river Verkeån primarily dominated by a uniform layer of gravel. Water changes were performed on the tanks between each experimental trial.

### 2.2 Animal Collection and Maintenance

On June 8, 2023, adult *U. crassus* were manually collected from the river Vramsån (55° 56′ 22.0″N 14° 09′ 55.1″E) with the aid of bathyscopes. Mussels were examined for gravidity by carefully opening them with specialized tongs and visually inspecting the gills for marsupial pouches (Beaver et al. 2019). A total of 48 non‐gravid and 6 gravid mussels (size range: 4.4–5.5 cm) were collected and transported to the MusselLAB in aerated tanks. In the MusselLAB, gravid mussels were kept in one aerated 10 L bucket, whereas non‐gravid mussels were introduced directly to the experimental tanks. Gravid mussels were monitored daily for an eventual glochidia release on June 20 (see Section 2.3). All mussels were starved for the duration of the study and returned to their home range at study termination.

Between May 31 and June 8, 2023, a total of 460 bullhead (mean length ± SD: 55.4 ± 8.1 mm) were electrofished from a 50 m stretch of the river Verkeån (55°42′38.3″N 13°59′17.9″E) with a flat DC, LUGAB L‐600 electrofishing unit (Bohlin et al. 1989). While this river lacks adult *U. crassus* , reintroductions of juvenile mussels have been carried out between 2021 and 2023 (LIFE Connects 2019). The captured bullhead were transferred to the MusselLAB in 70 L aerated buckets. On arrival, they were housed in three 200 L glass aquaria (100 × 40 × 50 cm) equipped with an external EHEIM (Classic 500) canister filter. Each aquarium had four perforated bricks to act as shelter and visual barriers for the bullhead and no other substrate. All tanks had 50% water changes performed daily. Bullhead were fed daily with frozen chironomid larvae until satiation. After infestation with *U. crassus* glochidia (see Section 2.3), the bullhead were housed in four separate tanks, two tanks for the infested fish and two for the non‐infested. After use in the habitat preference experiment, bullhead were housed in one of four additional tanks corresponding to each of the experimental treatments tested, treated as before. Bullhead from these trials were reused in the predation experiment, but were tested in a novel substrate size (see Section 2.4). After use in the final experiment, bullhead were sacrificed with an overdose of benzocaine to assess infestation intensity and to measure weight and length (ethical permit number: 001673—Göteborgs djurförsöksetiska nämnd).

Between June 8 and 9, 2023, approximately 1000 gammarids (mean length ± SD: 8.9 ± 3.3 mm) were collected from the river Vramsån (55°56′33.1″N 14°09′19.5″ E) and transported to the MusselLAB in a 40 L aerated bucket containing a variety of biotic river debris and some sand, later housed in a 70 L aerated bucket filled with the same material they were transported with. After use in the habitat preference experiment, gammarids were housed in a separate 70 L aerated bucket, set up as before, and reused in the predation study when necessary (see Section 2.4).

### 2.3 Infestation Procedure

On June 20, 2023, a glochidia release was observed in the gravid mussel bucket. The adult mussels were moved to a separate bucket and the water containing the glochidia was filtered through a 150 μm nylon mesh, later resuspended in 500 mL of clean water. Five 0.2 mL samples of glochidia suspension were taken and placed under a stereomicroscope to count the proportion of viable glochidia and to estimate total glochidia release. Glochidia viability was determined by adding a few grains of table salt (NaCl) to initiate a characteristic “snapping” stress reaction; this differentiated dead and living glochidia. We added 100 bullhead to a heavily aerated 20 L suspension of 95 living glochidia/L for 30 min. Compatibility between the bullhead and mussel populations used here has been previously determined through other work (LIFE Connects 2019). Overall infestation intensity was determined to be 9.1 ± 4.7 (mean ± SD) glochidia per fish between July 7 and 9, 2023 (17–19 days post infestation; dpi).

### 2.4 Experimental Methodology

Gammarid habitat preference was assessed between June 16 and 20, 2023 (*N* = 57). Four mussels (19 mussels/m^2^) were randomly arranged in one half of each experimental tank, after which 10 gammarids were released into the tank with one bullhead placed in a small cage in the sump of half the tanks to act as a source of predator cues (Figure 3A). The experimental tanks were then left overnight (~18 h). The morning after, a divider was placed between the mussel and non‐mussel sides of each tank to count how many gammarids were residing in each section. The bullhead was exchanged for each trial.

Bullhead habitat preference was assessed between July 4 and 7, 2023 (14–17 dpi; *N* = 40). This time during the infestation period was chosen to standardize possible effects of infestation on bullhead, as *U. crassus* typically excysts between 21 and 28 dpi (Schneider et al. 2017). As before, four mussels were randomly arranged in one half of each experimental tank and four bullhead (9.5 bullhead/m^2^), all either infested or not, were added to each of the experimental tanks (Figure 3B). The following day (~18 h), a divider was placed between the two mussel sections and bullhead position in either the mussel section or the non‐mussel section was counted.

Predation on gammarids by bullhead was assessed between July 7 and 9, 2023 (17–19 dpi; *N* = 34). Different from before, eight mussels were arranged evenly over the entire bottom of half of the experimental tanks (19 mussels/m^2^). Ten gammarids were added to each tank and allowed to find refuge for 30 min, after which four bullhead of either infestation condition were added to the tank (Figure 3C). As before, the tanks were left overnight (~18 h) for inspection the morning after. Here, bullhead were immediately removed from the tank and sacrificed with an overdose of benzocaine. Bullhead were then measured and dissected to assess body size and infestation success. The remaining gammarids in each tank were then counted to evaluate bullhead predation rate.

In all cases, catching and counting the study animals required removing the gravel and cobbles; this also allowed for the removal of any pseudofeces produced by the mussels. The amount of pseudofeces produced by the mussels in this study was not quantified, but was visually confirmed on and around the mussels in every trial. Gammarids and bullhead were not observed interacting with the pseudofeces in this study. All treatment conditions were randomized between experimental days with one noteworthy exception: the predator cue condition was not randomized between tanks to ensure no lingering cue in the experimental tank, as there was not enough processed water available to wash each tank between trials.

Not all gammarids were recovered in all trials on gammarids habitat preference (24% of trials). To compensate for this, the proportion of gammarids counted in the mussel section over the total number of gammarids recovered was used rather than the overall number of gammarids on the mussel side. In both the gammarid and bullhead habitat preference experiments, mussels occasionally moved within the tanks such that they could not be divided into two even sections; these replicates were removed from the final data set.

### 2.5 Statistical Analysis

A Linear Mixed Model was fitted to the proportion of gammarids recovered on the mussel side using substrate size (gravel/cobble), predator cues (yes/no), and the substrate × predator cue interaction as fixed factors. Tank ID was additionally nested within the predator cues × substrate size interaction as a random factor to account for the lack of randomization of this variable. One‐sample t‐tests were used to compare gammarid preference for mussel‐dominated habitats against random chance habitat selection (0.5 proportion of gammarids on the mussel side). The significance level was adjusted with a Bonferroni correction (four tests; significance level = 0.0125).

A linear model was fitted to the number of bullhead counted on the mussel side of the experimental tank (*bullhead with mussels*), using substrate size (gravel/cobble), infestation (yes/no) and the substrate × infestation interaction as fixed factors. As with Gammarid habitat choice, one‐sample t‐tests were used to compare bullhead preference for mussel‐dominated habitats against random chance habitat selection (2 bullhead on the mussel side). The significance level was adjusted with a Bonferroni correction (four tests; significance level = 0.0125). A second linear model was fitted to the number of gammarids recovered after the predation period (*gammarid survival*) using substrate size (gravel/cobble), infestation (yes/no) and mussel presence (yes/no) and all combinations of two‐way and three‐way interaction effects as fixed factors (see Table 1 for a complete list of all factors in the model).

**TABLE 1:** Degrees of freedom (DF), F‐statistic, and significance level of factors used in the general linear models for bullhead habitat preference, gammarid habitat preference, and gammarid survival.

Experiment	Factor	DF	*F*	*p*
Gammarid habitat preference	Tank ID (Predator cue × Substrate)	8, 45	0.533	0.825
	Substrate size	1, 8.2	0.201	0.666
	**Predator cue**	**1, 8.2**	**7.761**	**0.023**
	Substrate size × Predator cue	1, 8.2	0.298	0.600
Bullhead habitat preference	Substrate size	1, 36	3.355	0.075
	Infestation	1, 36	0.063	0.803
	Substrate size × Infestation	1, 36	1.237	0.274
Gammarid survival	**Substrate size**	**1, 26**	**19.978**	**< 0.001**
	Infestation	1, 26	1.367	0.253
	**Mussel presence**	**1, 26**	**5.360**	**0.029**
	Substrate size × Infestation	1, 26	0.232	0.634
	**Substrate size** × **Mussel presence**	**1, 26**	**6.242**	**0.019**
	Infestation × Mussel presence	1, 26	0.442	0.512
	Substrate size × Infestation × Mussel presence	1, 26	0.013	0.910

*Note:* Significant factors are shown in bold.

## 3 Results

**FIGURE 4:**
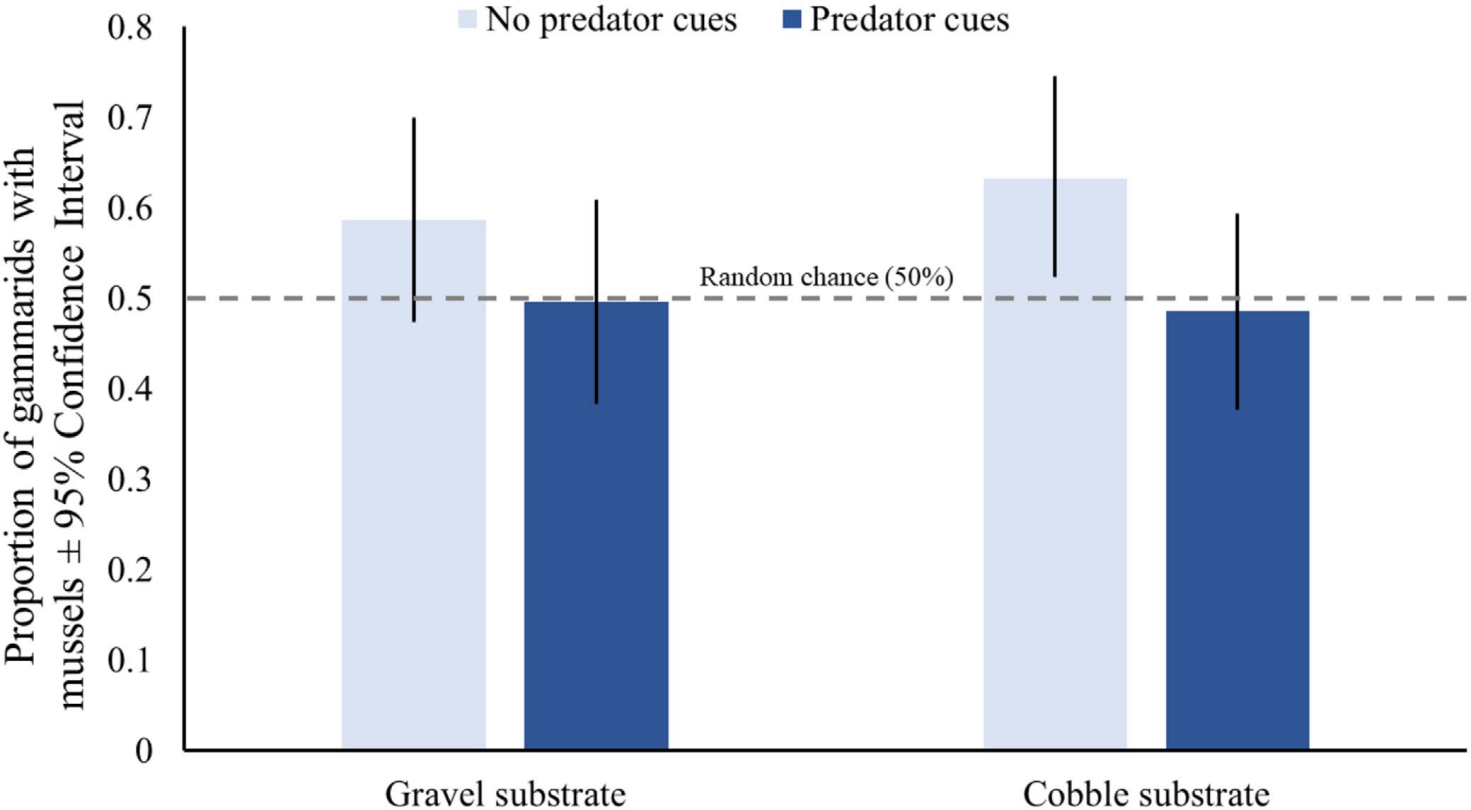
Proportion of gammarids ( *G. pulex* ) with mussels ( *U. crassus* ) in different habitat types. Gammarids chose mussel habitats at a significantly higher rate in the absence of predator cues; when predator cues were present, gammarids chose habitats randomly. Gammarids only chose mussel habitats at a rate significantly different from random chance in the cobble‐dominated substrate. The dashed line indicates threshold for random chance (0.5 proportion of gammarids on the mussel side; 50%).

In the absence of predator cues, gammarids chose the mussel‐dominated habitats significantly more often than in the presence of predator cues (5.9 ± 0.4 vs. 4.7 ± 0.4; mean number of gammarids with mussels ± SE), a behavior that was not significantly affected by substrate size or the substrate × predator cue interaction term (Table 1). Gammarids were recovered in mussel‐dominated habitats at rates above random chance in the cobble‐dominated substrate but not in the gravel‐dominated substrate in the absence of predator cues (Figure 4; Table 2). Tank ID did not significantly affect gammarid preference for mussel‐dominated habitats (Table 1).

**TABLE 2:** Sample size (*n*), test statistic (*t*), and significance level (*p*) of gammarid and bullhead habitat preference against random chance habitat selection.

Experiment	Substrate	Secondary treatment	*n*	*t*	*p*
Gammarid habitat preference	Gravel	No predator cues	14	1.572	0.0699
		Predator cues	15	−0.086	0.4662
	Cobble	**No predator cues**	**14**	**2.557**	**0.0119**
		Predator cues	14	−0.123	0.4519
Bullhead habitat preference	Gravel	Non‐infested	9	−0.426	0.3405
		Infested	11	−1.000	0.1704
	Cobble	Non‐infested	10	0.361	0.3632
		Infested	10	2.236	0.0261

*Note:* Results significant under the Bonferroni corrected level (*p* < 0.0125) are reported in bold.

**FIGURE 5:**
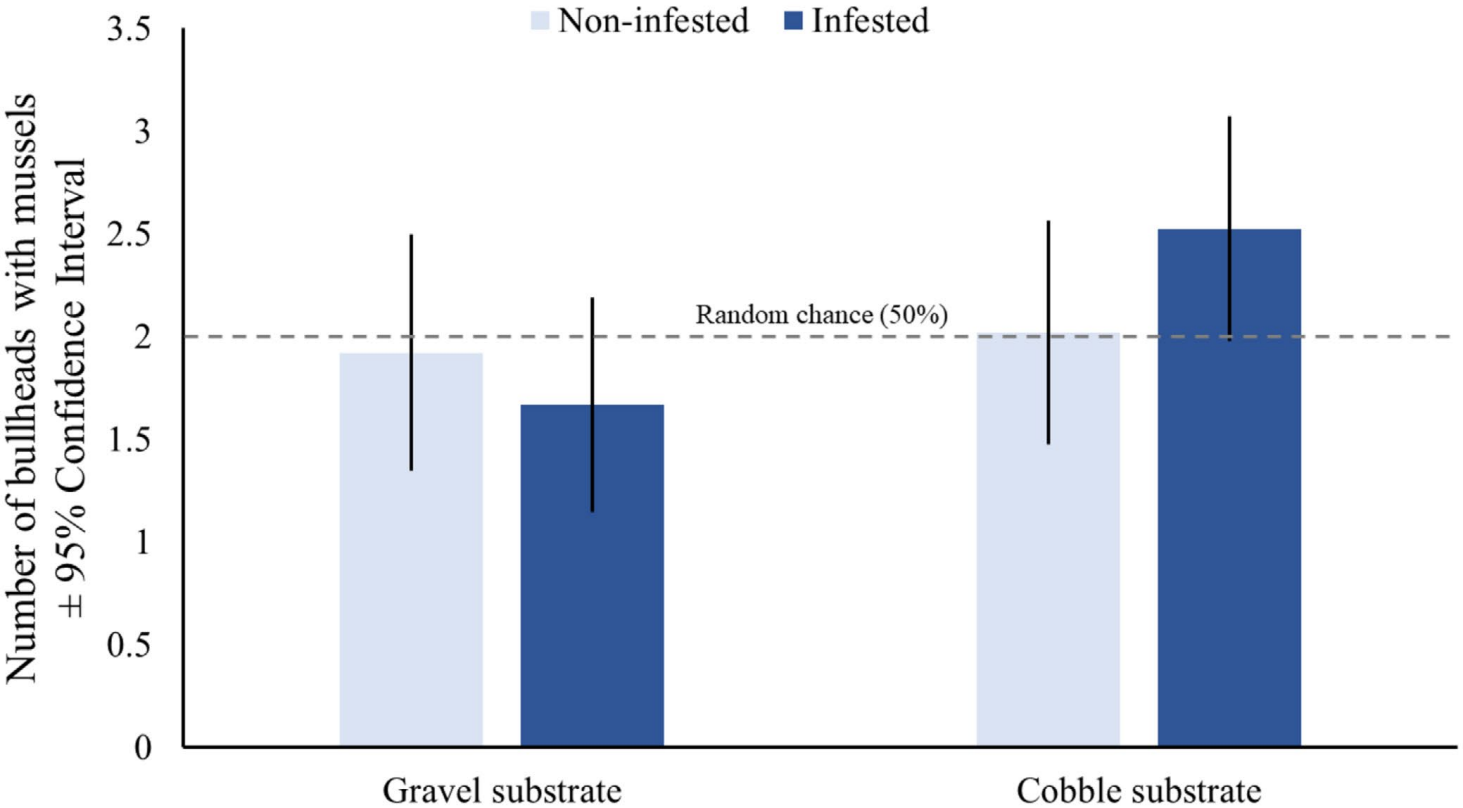
Number of bullhead ( *C. gobio* ) with mussels ( *U. crassus* ) in different habitat types. Bullhead did not differ in their preference for mussel habitats between substrate sizes or when infected with glochidia from *U. crassus* . Infested bullhead had a near‐significant preference for mussel habitats when infested with glochidia from *U. crassus* when in the cobble‐dominated substrate. Dashed line indicates threshold for random chance (2 bullhead on the mussel side; 50%).

Infested bullhead did not differ significantly from non‐infested bullhead in their preference for mussel‐dominated habitats. Neither substrate size nor the substrate size × infestation term significantly impacted bullhead habitat preference, though substrate size did approach significance, indicating a possible bullhead preference for mussel‐dominated cobble substrates (Table 1). In addition, in the cobble‐dominated substrate, infested bullhead were recovered in mussel‐dominated habitats at a rate near significantly different from random chance (Figure 5; Table 2).

**FIGURE 6:**
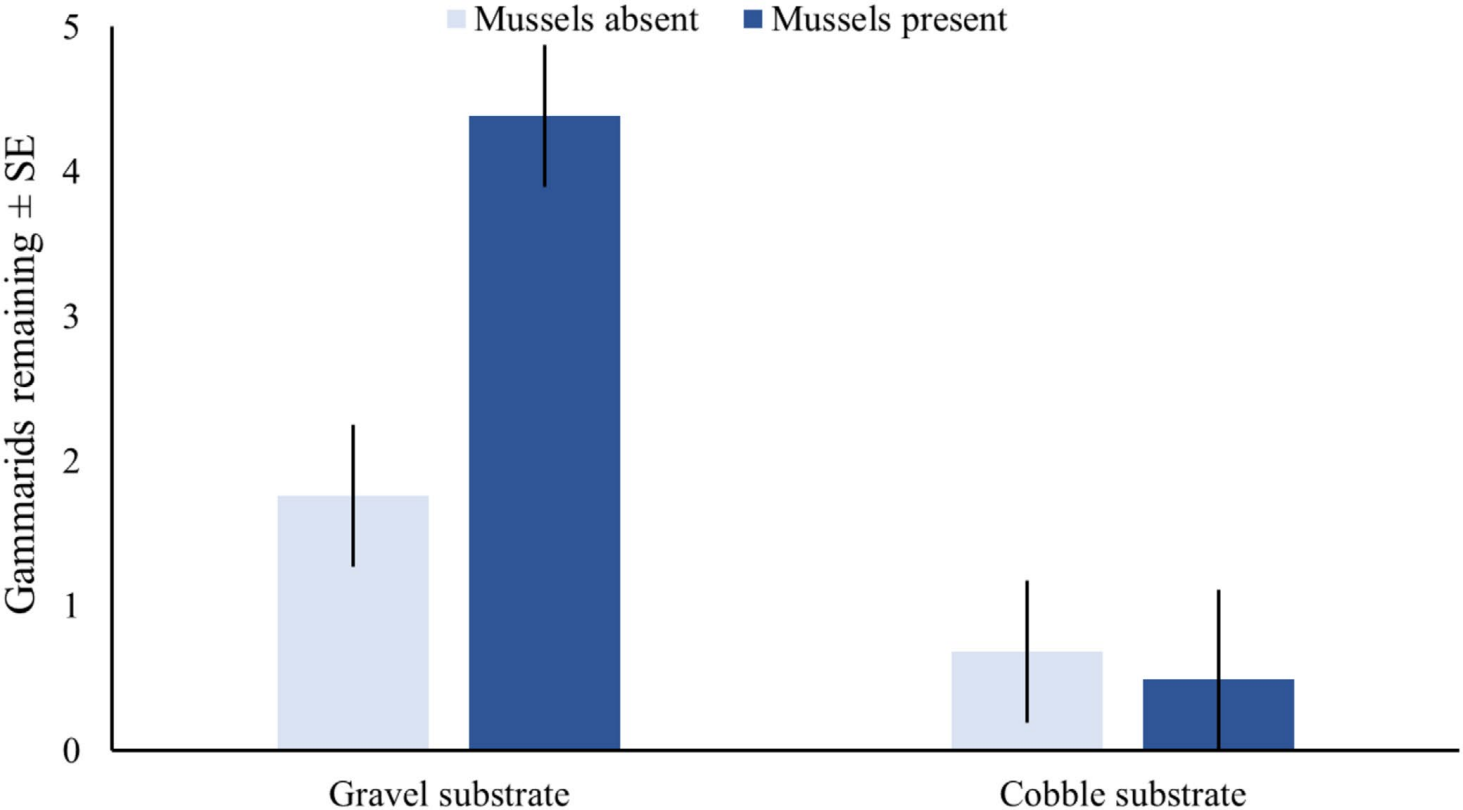
Number of gammarids ( *G. pulex* ) remaining after a period of predation by bullhead (*C. gobbio*) when infested with glochidia from ( *U. crassus* ) in different habitat conditions when adult *U. crassus* were present or absent.

In the absence of mussels, gammarid survival under bullhead predation was three times higher in the gravel‐dominated substrate (1.8 ± 1.6 gammarids recovered ± SE) compared to the cobble‐dominated substrate (0.7 ± 1.0 gammarids recovered ± SE). When mussels were present in the gravel‐dominated substrate, survival was additionally increased approximately three times over (4.4 ± 2 gammarids recovered ± SE; Figure 6). Gammarid survival under bullhead predation was significantly impacted by substrate size, mussel presence, and the interaction between the two (Table 1). Infestation, and the interaction of infestation with the other factors, did not significantly impact gammarid survival.

## 4 Discussion

The results presented here demonstrate that adult freshwater mussels have the potential to alter predator–prey interactions, highlighting the importance of studies on trophic interactions. We demonstrate that adult *U. crassus* impacted the habitat choice of both gammarids and infested bullheads, particularly in cobble‐dominated substrates, attracting both species to mussel beds. This indicates that mussel beds may significantly increase predator–prey interactions. In addition, adult mussels have the potential to increase their own reproductive success, as recently metamorphosed juvenile mussels may predominantly excyst around adults—a habitat we may assume to be suitable for the new mussel generation (Uryu et al. 1996; Irmscher and Vaughn 2018). Interestingly, gammarids chose mussel‐dominated habitats less in the presence of predator cues, despite having increased survival in those habitats. Together, these results highlight mechanisms by which freshwater mussels can impact the interactions of their surrounding benthic community, underscoring their importance as ecosystem engineers.

Gammarid species sympatric with specific mussel species (e.g., *Dikerogammarus villosus* and *Dreissena polymorpha* ) preferentially choose mussel beds, using them as a source of habitat, refuge from predators and nutrients (Kobak and Żytkowicz 2007; Gergs and Rothhaupt 2008a, 2008b; Kobak et al. 2014). Here, we show that a gammarid species sympatric to *U. crassus* demonstrates a similar, though marginal, interaction; preferentially selecting mussel habitat where they have higher survival, suggesting that in the wild *U. crassus* may act as habitat for gammarids. However, our gammarids did not prefer mussel habitats in the presence of acute predator cues (tested here). As gammarids generally avoid feeding and seek shelter in the presence of acute predator cues, we propose that our mussels may provide an alternative resource to our gammarids in the form of egested mucus or pseudofeces produced by our mussels (Dahl and Greenberg 1996; Åbjörnsson et al. 2000; Harrison et al. 2005; Ahlgren et al. 2011; Jermacz and Kobak 2017; Beermann et al. 2018). We propose this as the material egested by mussels represents the only organic material available for gammarids to feed on during the trials. If we assume gammarids fed on material produced by mussels in our study, the results presented here would be in line with previous study; with gammarids congregating around a source of nutrients in the absence of predators, and avoiding nutrient‐rich areas in the presence of acute predator cues (e.g., Jermacz and Kobak 2017; Beermann et al. 2018). Regardless of the speculative interaction between gammarids and mussel mucus and pseudofeces here, our results suggest that gammarids may congregate around mussel beds.

Infested bullhead showed no significant preference for mussel habitats nor differences in predation compared to non‐infested individuals. However, infested bullhead showed a tendency to prefer mussel habitats in cobble‐dominated substrates, suggesting that infestation may affect habitat preference in cobble substrates; an effect we propose given the combined near‐significant effect of substrate size and the near‐significantly different habitat choice from random chance. The infestation rate used here (9 glochidia per fish) is comparable to natural levels, but can be an order of magnitude higher without causing significant mortality (Douda et al. 2012; Lamand et al. 2016; Schneider et al. 2017). Glochidiosis typically reduces host swimming performance and feeding ability, with the effects often magnifying with infestation intensity and around the period of mussel excystment (Crane et al. 2011; Taeubert and Geist 2013; Österling et al. 2014; Filipsson et al. 2016; Rock et al. 2025). Had the infestation rate been higher, or had the habitat preference test been run during the period of juvenile mussel excystment, the effect of infestation on bullhead habitat preference would have likely been more pronounced (Rock et al. 2022). In such light, our results may lend some support to previous proposals of host manipulation by unionid mussels, and suggest that the ecosystem functions of the adult life stage may play a role in this manipulation (Rock et al. 2025). We suggest that the increased habitat complexity provided by adult mussels, an effect more prominent in the cobble substrate than the gravel substrate, may benefit the next generation of mussels. Encysted glochidia do not gain a fitness advantage by reducing host feeding performance while simultaneously extracting nutrients from their hosts, as premature host death would eliminate encysted glochidia (Taeubert et al. 2012; Denic et al. 2015), an effect we observe (i.e., no significant difference in feeding rates). However, those same glochidia would gain a fitness advantage by reducing host swimming performance, and attracting infested hosts to habitats with higher structural complexity (i.e., mussel‐dominated cobble substrates) with lower water velocities more suitable for juvenile mussels after excystment (Smokorowski and Pratt 2007; Sansom et al. 2018, 2022; Hopper et al. 2019; Rock et al. 2025). It is not possible to distinguish between an advantageous product of selection or an advantageous by‐product of parasitism from glochidia; regardless, the effect on host ecology remains (Poulin 2010).

Gammarids had significantly higher survival in the gravel‐dominated substrate compared to the cobble‐dominated substrate, likely due to the increased habitat complexity of the permeable gravel bed versus the bare sand bottom of the cobble‐dominated substrate. Mussel presence did not improve gammarid survival in the cobble‐dominated substrate, possibly because the mussel density used here did not suffice to significantly enhance habitat complexity. Mussel presence has been shown to reduce predation rates of gammarids by bullhead (Beekey et al. 2004; Kobak et al. 2014; Coughlan et al. 2022). However, the effect of mussel presence on gammarid survival in the gravel‐dominated substrate was likely not solely due to increased habitat complexity as bullhead habitat preference was unaffected by mussel presence in gravel‐dominated substrates, nor did the mussel density increase gammarid survival in the cobble‐dominated substrate. The reasons for this effect cannot be determined by this study; thus, we speculate the following hypothesis: Although not directly observed, we hypothesize that bullhead may have occasionally fed directly on mussel pseudofeces and other egested mucus in the gravel substrate where gammarids were less readily available, increasing satiation and reducing hunting behavior. Unionids have developed various host attraction mechanisms to aid glochidia transmission, developed from fish ingesting material egested by gravid mussels containing glochidia (Haag and Warren Jr 2003; Klunzinger et al. 2023). Non‐reproductive mussels have been documented to use reward mimicry (Jamie 2017), attracting fish with nutrient‐rich material to improve the reproductive success of females who attract host fish with lures (Jones et al. 2023). *U. crassus* is one of only two European species with known host attraction behavior, though without mantle displays (Aldridge et al. 2023; Rock 2024). Further research is needed to validate if bullheads occasionally ingest pseudofeces, and to explore the presence of additional host attraction mechanisms by *U. crassus* .

Our suggestion that both gammarids and bullhead occasionally feed on material egested from *U. crassus* is speculative and requires a dedicated investigation. Notwithstanding, our results indicate that infested host behavior may be altered in a manner beneficial to juvenile mussels, which may support previous suggestions that unionid mussels express an extended phenotype and manipulate host behavior. The results presented here demonstrate that freshwater mussels can have significant impacts on the predator–prey interactions between their hosts and their prey, beyond simply increasing habitat complexity, opening the possibility for currently undescribed species interactions. As the populations of freshwater mussels continue to decline so too will their impacts on the broader aquatic ecosystem across multiple trophic levels. As more conservation measures are implemented to address the decline in freshwater mussel populations, so too should the associated research on their broader community‐wide impacts, both as adults and as parasites, to better understand the community‐wide ramifications in their conservation.

## Author Contributions

**Sebastian L. Rock:** conceptualization (equal), data curation (equal), formal analysis (equal), investigation (equal), methodology (equal), resources (equal), visualization (equal), writing – original draft (equal). **Anna M. Elmlund:** investigation (equal), methodology (equal), writing – review and editing (equal). **P. Anders Nilsson:** conceptualization (equal), formal analysis (equal), methodology (equal), project administration (equal), supervision (equal), writing – review and editing (equal). **Johan Watz:** conceptualization (equal), formal analysis (equal), methodology (equal), project administration (equal), supervision (equal), writing – review and editing (equal). **Olle Calles:** funding acquisition (equal), project administration (equal), supervision (equal), writing – review and editing (equal). **Martin Österling:** conceptualization (equal), funding acquisition (equal), project administration (equal), supervision (equal), writing – review and editing (equal).

## Funding

This work was supported by the EU LIFE Program (Project acronym: LIFE CONNECTS; LIFE18 NAT/SE/000742), Karlstad University, and Lund University.

## Conflicts of Interest

The authors declare no conflicts of interest.

## Supporting information


**Data S1:** ece372601‐sup‐0001‐DataS1.xlsx.

## Data Availability

The data that supports the findings of this study is available at the following link: https://doi.org/10.5061/dryad.44j0zpcvf.
